# The miR-183 family cluster alters zinc homeostasis in benign prostate cells, organoids and prostate cancer xenografts

**DOI:** 10.1038/s41598-017-07979-y

**Published:** 2017-08-09

**Authors:** Shweta Dambal, Bethany Baumann, Tara McCray, LaTanya Williams, Zachary Richards, Ryan Deaton, Gail S. Prins, Larisa Nonn

**Affiliations:** 1 Department of Pathology, University of Illinois at Chicago, Chicago, IL 60612 USA; 2 Department of Urology, University of Illinois at Chicago, Chicago, IL 60612 USA; 3 University of Illinois Cancer Center, Chicago, IL 60612 USA

## Abstract

The miR-183 cluster, which is comprised of paralogous miRs-183, -96 and -182, is overexpressed in many cancers, including prostate adenocarcinoma (PCa). Prior studies showed that overexpression of individual pre-miRs-182, -96 and -183 in prostate cells decreased zinc import, which is a characteristic feature of PCa tumours. Zinc is concentrated in healthy prostate 10-fold higher than any other tissue, and an >80% decrease in zinc is observed in PCa specimens. Here, we studied the effect of overexpression of the entire 4.8 kb miR-183 family cluster, including the intergenic region which contains highly conserved genomic regions, in prostate cells. This resulted in overexpression of mature miR-183 family miRs at levels that mimic cancer-related changes. Overexpression of the miR-183 cluster reduced zinc transporter and intracellular zinc levels in benign prostate cells, PCa xenografts and fresh prostate epithelial organoids. Microarray analysis of miR-183 family cluster overexpression in prostate cells showed an enrichment for cancer-related pathways including adhesion, migration and wound healing. An active secondary transcription start site was identified within the intergenic region of the miR-183 cluster, which may regulate expression of miR-182. Taken together, this study shows that physiologically relevant expression of the miR-183 family regulates zinc levels and carcinogenic pathways in prostate cells.

## Introduction

The peripheral zone of the prostate accumulates the highest levels of zinc of any soft tissue in the human body^1^. Consequently, high concentrations of zinc in the prostate epithelium inhibit aconitase enzyme activity leading to a buildup of citrate, which is then secreted into the prostatic fluid^1–3^. In contrast, prostate cancer (PCa) lesions have reduced zinc and citrate concentrations that are approximately 80% lower than benign prostate^4–7^.

Cellular zinc homeostasis is regulated by fourteen ZIP (SLC39A) and ten ZNT (SLC30A) zinc transporters, which are present on the cell membrane and the membranes of intracellular organelles^5, 8, 9^. ZIP transporters (Zrt-Irt-like Proteins) increase cytosolic zinc levels via extracellular import and export from organelles. Conversely, ZNT transporters decrease cytosolic zinc. Altered zinc homeostasis may be permissive for PCa development, as zinc regulates crucial pathways involved in carcinogenesis including proliferation, apoptosis, and cellular metabolism^3, 10, 11^. In PCa cells, zinc inhibits proliferation by blocking the G2/M cell cycle check point^12^, and is pro-apoptotic by several mechanisms including increased Bax/BCL-2 ratio^13^ and decreased NF-κB leading to caspase 3/7 activation^14^. Of all the zinc transporters, ZIP1 is the major zinc transporter in the prostate epithelium^15^, and ZIP1 levels are lower in malignant prostate lesions compared to benign tissue^5^. ZIP1 has tumour-suppressive properties, as overexpression of ZIP1 in RWPE-2 PCa cells decreased proliferation and increased apoptosis^16^. As well, *in vivo*, ZIP1-overexpressing PC3 xenografts had reduced tumour size, reduced NF-κB signalling and increased apoptosis^17^. Together these studies provide support for tumour suppressive activity for both zinc and ZIP1 in the human prostate.

Our lab previously demonstrated that ZIP1 expression and zinc homeostasis were regulated by the microRNAs (miRs)-96, -82 and -183, which comprise the miR-183 cluster family^18^. These miRs are present at higher levels in prostate cancer tissue compared to benign epithelium^18^ and overexpressed in the majority of cancer types^19^. Ectopic expression of the pre-miRs-183, -96 and -182 decreased ZIP1 mRNA and protein, and functionally reduced zinc import into benign prostate epithelial cells^18^. The human miR-183 family members are found in a cluster on chromosome 7q32 with adjacent miRs-183 and -96 separated by a ~4 kb intergenic region from miR-182, making the pri-microRNA 4.8 kb in length. The sequences of the miR-183 family miRs and presence of the intergenic region are highly conserved, indicating an evolutionary advantage to retaining this miR cluster^19^.

A limitation of our original study, and other reports of the miR-183 family, is that very high levels of the miRs are achieved by the use of pre-miRs, therefore the phenotypes may not have physiological relevance to cancer. As well, we have observed that mature miRs-183, -96 and -182 are differentially expressed in prostate cells^18^, suggesting potential regulation via sites within its intergenic region. In this study, we created a lentiviral-based overexpression system of the miR-183 cluster family, which included the entire 4.8 kb genomic sequence with the intergenic region, to better mimic cancer-related changes and to achieve physiologically relevant levels of the miRs in prostate cells. The miR-183 cluster was overexpressed in benign prostate cells, fresh human prostate epithelial organoids and in PCa cell xenografts *in vivo*. The regulation of cancer-related pathways by the miR-183 family cluster was also examined by microarray gene expression analysis.

## Results

### Physiologically relevant overexpression of the miR-183 cluster in benign prostate epithelial cells

**Figure 1:**
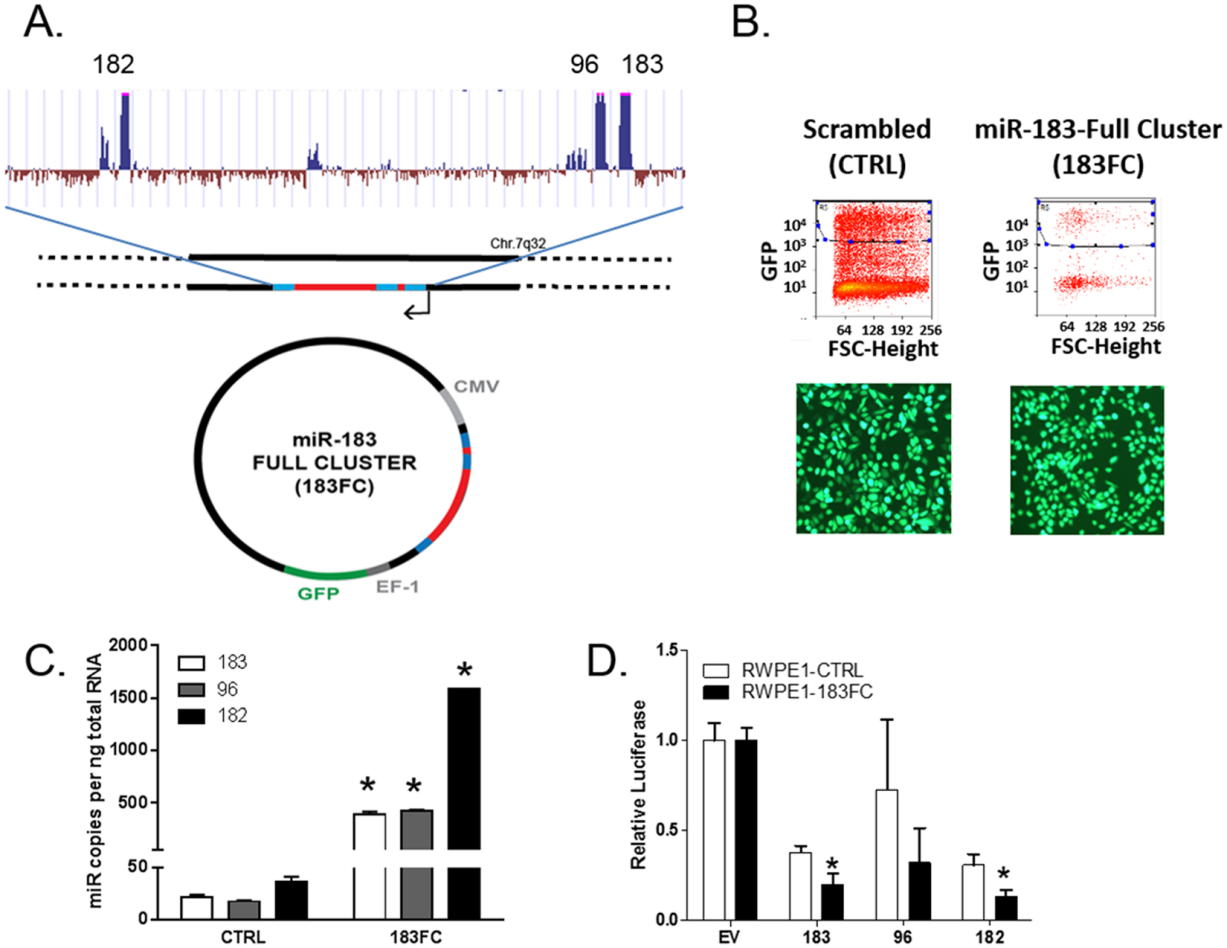
Overexpression of the miR-183 family cluster in benign prostate epithelial cells. (**A**) The gene for the miR-183 family cluster was isolated from male genomic DNA using nested PCR and cloned into CD511B-1 Lentiviral plasmid. (**B**) Sorting of GFP+ RWPE1-CTRL and RWPE1-183FC cells for homogeneous polyclonal population. (**C**) Expression of mature miRs-183, -96 and -182 by RT-qPCR in RWPE1 cells. Absolute quantitation from a standard curve and normalized to RNU44. Mean of N = 6 shown with SEM. (**D**) luciferase levels of plasmids containing the miR consensus sequences in the 3′ UTR transiently transfected in RWPE1-CTRL and RWPE1-183FC. Graphs show mean data −/+ SEM (n = 2) relative to empty vector without miR sites in the 3′ UTR (EV). *p < 0.05 CTRL versus 183FC by Student’s paired 2-sided t-test.

In order to assess the functional effects of the miR-183 family cluster when expressed from its endogenous genomic sequence at physiologically relevant expression levels, the entire 4.8 kb miR-183 cluster genomic region containing miRs-183, -96 and -182 (miR-183 full cluster or 183FC) was cloned into CD511B-1, a lentiviral vector with a GFP tracer (Fig. 1A). Transduction and GFP^+^ selection (Fig. 1B) of benign prostate epithelial RWPE-1 cells with CD511B-183FC (RWPE1-183FC) resulted in 10–30 fold overexpression of the miR-183 cluster members compared to CD511B-CTRL transduced cells (RWPE1-CTRL) population (Fig. 1C). These levels are similar to those observed in prostate cancers compared to benign prostate^18^. Of note, the expression levels were determined by absolute quantitation and expression of the three miRs was not uniform, suggesting differential regulation of the three miRs. Transfection with luciferase constructs containing miR consensus sites in the 3′ UTR validated that the overexpressed miRs were functional (Fig. 1D).

### ZIP1 repression and reduced total and accessible zinc in RWPE1-183FC cells

**Figure 2:**
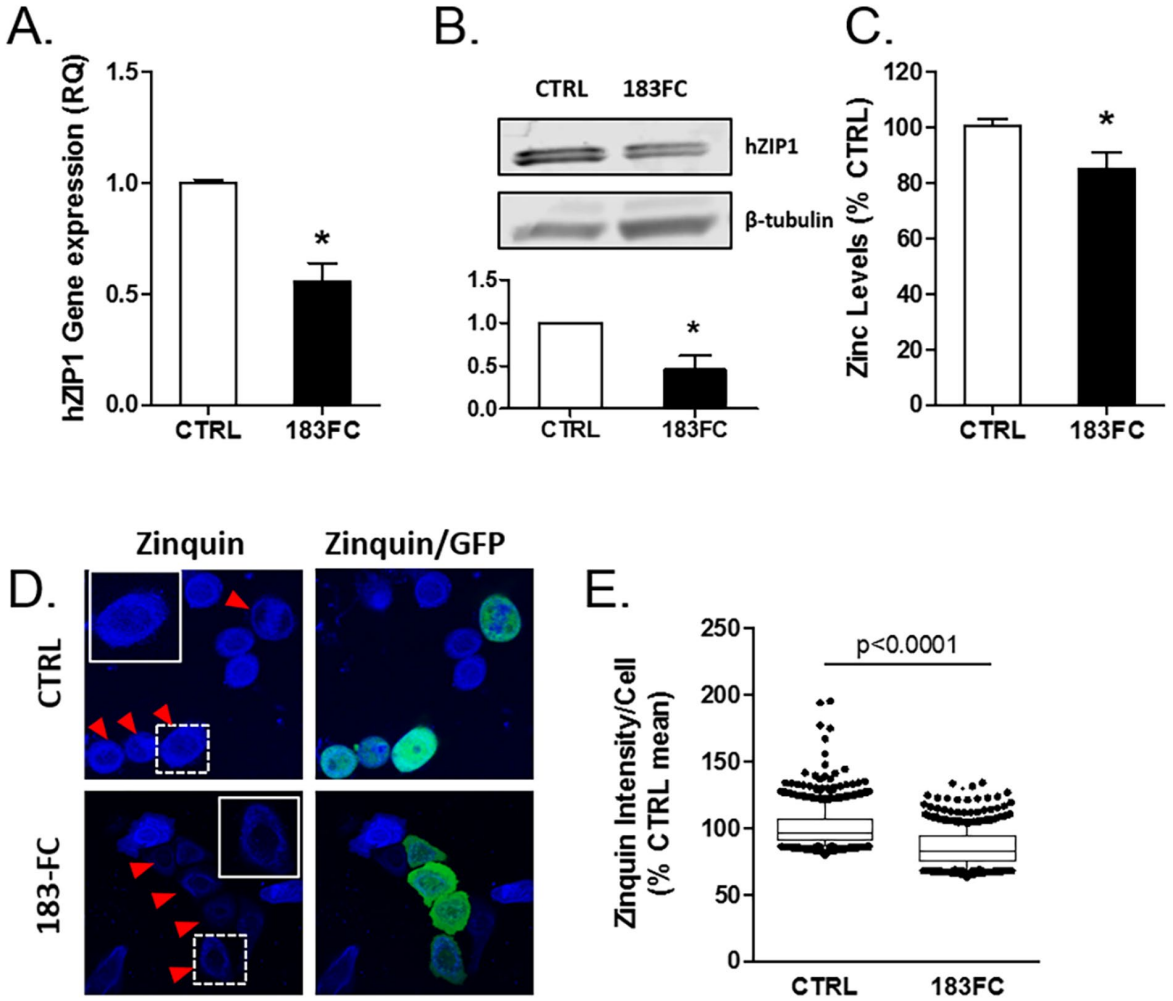
ZIP1 mRNA, hZIP1 protein and zinc levels were lower in miR-183-FC cells. (**A**) hZIP1 mRNA gene expression measured by RT-qPCR in RWPE1-CTRL and RWPE1-183FC cells. (**B**) ZIP1 protein levels measured by immunoblot and quantified. (**C**) Chromogenic zinc assay in RWPE1-CTRL and 183FC cells (n = 3 experiments). Mean shown with SEM. (**D**) Zinquin (blue) fluorescence in RWPE1-CTRL and RWPE1-183FC cells was imaged with confocal microscopy. Naïve RWPE1 cells were mixed with GFP+ cells. Arrows indicate GFP+ transduced RWPE1 cells. (**E**) Zinquin intensity quantified per cell by inForm™ image analysis in RWPE1-CTRL and RWPE1-183FC cells. N = 3 experiments were analyzed as percentage of CTRL mean per experiment. Data from all 3 experiments were analyzed together by Mann-Whitney nonparametric test and are shown as boxplots with mean (line), 25–75% (box) and 95% confidence intervals (whiskers) and individual cell outliers. *p < 0.05 by Mann-Whitney nonparametric test for non-normal distribution of data in parts A and E, Student’s paired 2-sided t-test for parts **B** and **C**.

The ZIP1 3′ UTR contains two validated binding sites for the miR-183 family^18^. A 50% and 53% reduction in the levels of ZIP1 mRNA and protein, respectively, was observed in the RWPE1-183FC cells compared to RWPE1-CTRL (Fig. 2A,B). Total zinc levels were reduced significantly by 15% in RWPE1-183FC cells compared to RWPE1-CTRL by a chromogenic assay (Fig. 2C).

The spatial distribution of accessible zinc was assessed with Zinquin ethyl ester (ZQ), a fluorescent cell-permeable zinc probe that becomes trapped within the cell after cleavage of the ethyl ester moiety by cytosolic esterases^20^. Naïve RWPE-1 cells were used as a control and were mixed 1:1 with the GFP^+^ RWPE1-CTRL or RWPE1-183FC cells. Confocal microscopy showed a perinuclear and cytoplasmic distribution of zinc that was dramatically reduced in the GFP^+^ RWPE1-183FC cells compared to naive RWPE-1 cells (Fig. 2D), whereas ZQ in the GFP^+^ RWPE1-CTRL cells was similar to the naïve cells. Digital image quantification of individual cells showed that ZQ intensity was significantly lower (*p < 0.0001) in the RWPE1-183FC cells compared with the RWPE1-CTRL GFP^+^ cells (Fig. 2E). These data show that miR-183 cluster overexpressing cells have reduced levels of intracellular zinc. Notably, the zinc phenotype is striking in the ZQ study as ZQ binds to free (labile), loosely-bound zinc, and accessible zinc^20^.

### Primary human epithelial prostate organoids with 183FC had diminished zinc by x-ray fluorescence

**Figure 3:**
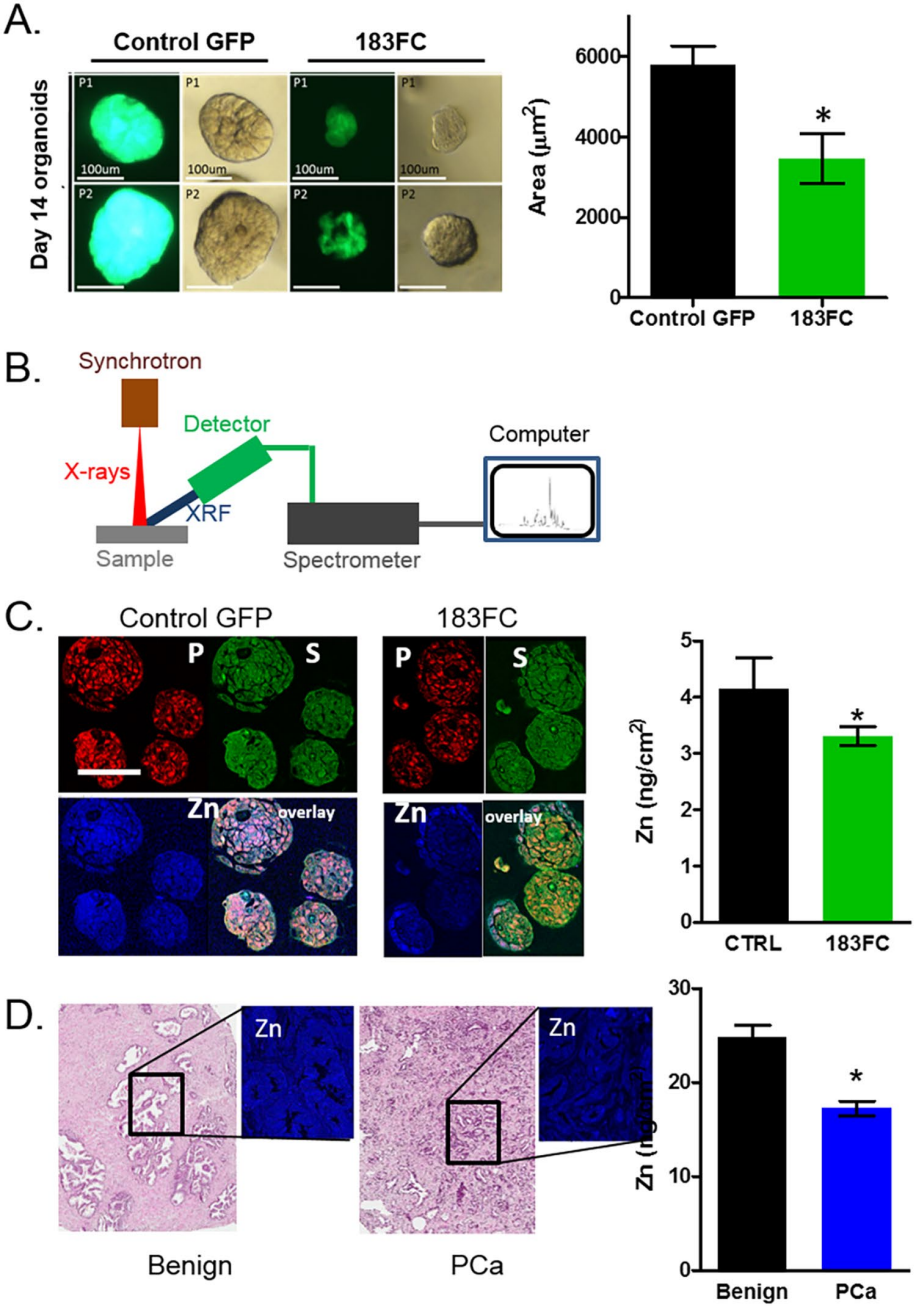
Overexpression of 183FC in benign human prostate epithelial organoids emulated decrease in zinc observed in human PCa as measured by X-ray fluorescence (XRF). (**A**) Size of 14 day organoids transduced with control-GFP or 183FC. Two individual PrE patient-derived cell lines are shown (P1 and P2) of n = 4 total patients. (**B**) Schematic of x-ray fluorescence measurement at Argonne National Lab (full detail in Supplemental Fig. 1). (**C**) Images and quantitation of the fluorescence of the elements sulfur (S), phosphorus (P), and zinc (Zn) in 14 day benign organoids (n = 4) transduced with control-GFP or 183FC scanned with x-rays. Zinc levels were quantified by ROI’s drawn to encompass the entire organoid. Graphs show mean zinc per area of each of the organoids. (**D**) H&E image and quantitation of the fluorescence of zinc (Zn) in benign and PCa patient tissue scanned with x-rays. Quantitation based on 10 ROIs for each tissue. All graphs show mean with SEM, * < 0.05 by Student’s unpaired 2-sided t-test.

Primary human prostatic epithelial cells (PrE), derived in our lab, were used as a second *in vitro* preclinical model to assess zinc regulation by 183FC. Following lentiviral infection, single cell PrE cells were cultured in matrigel for 14 days to form prostate organoids (Fig. 3 and Supplemental Fig. 1). 183FC organoids were markedly smaller than the GFP controls (Fig. 3A). Total zinc was assessed by X-ray fluorescence (Fig. 3B,C and Supplemental Fig. 1) and was lower in 183FC organoids. Notably, the 183FC organoids lacked zinc in the differentiated cells in the centres of the organoids (Fig. 3C). This reduction in zinc was similar in magnitude to the reduction of zinc in PCa tissue compared to benign patient tissue by the same method (Fig. 3D).

### *In vivo* reduction in intra-tumoural zinc and increase of tumor volume in RWPE2-183FC xenografts

**Figure 4:**
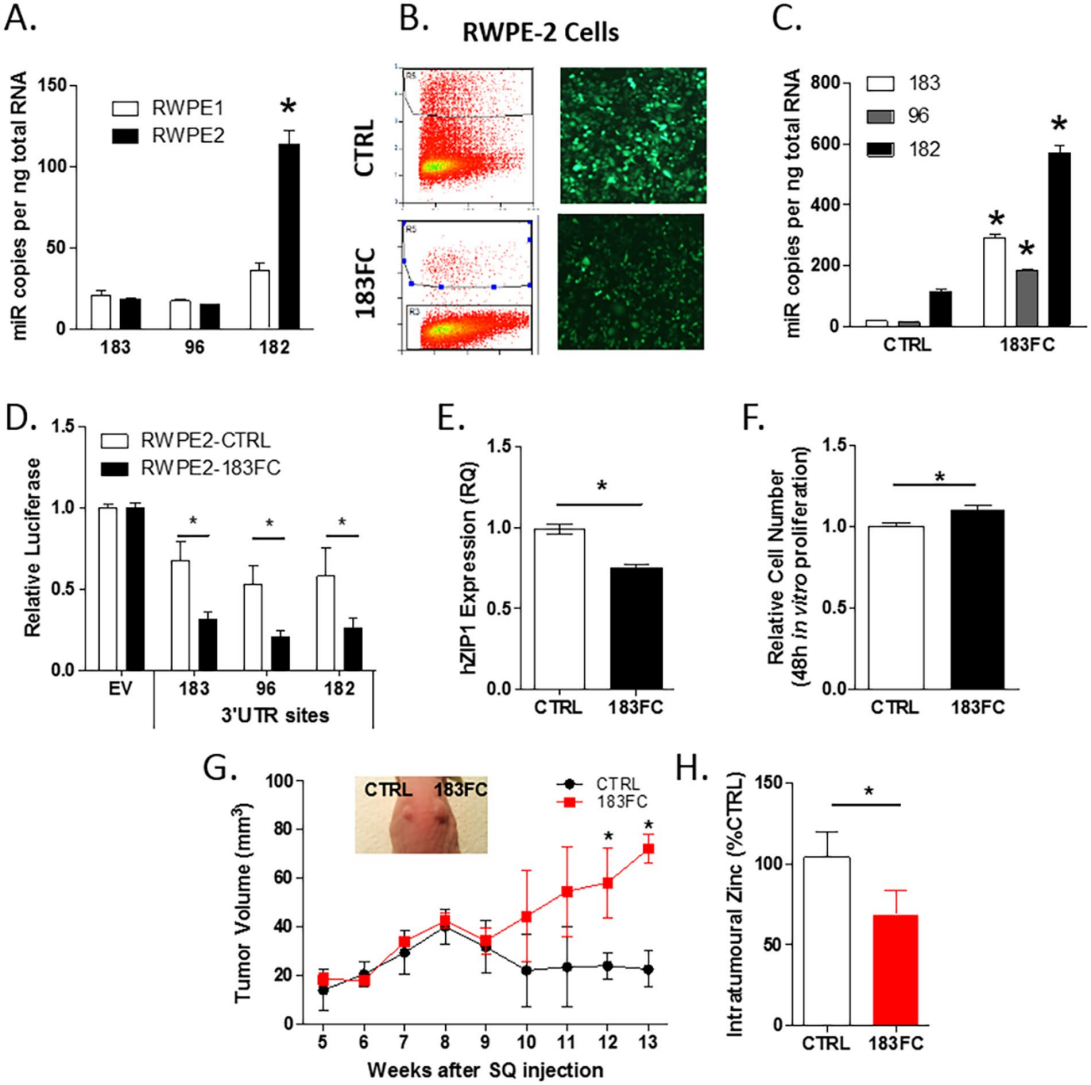
miR-183-FC expression lowered intra-tumoural zinc and increased tumour size *in vivo*. (**A**) Expression of miRs-183, 96 and 182 in RWPE2 prostate cancer cells compared to benign RWPE1 by RT-qPCR. Relative quantity to RWPE1 cells and normalized to RNU44. (**B**) RWPE2 cells were transduced with CTRL or 183FC lentivirus and FACS sorted. (**C**) Absolute quantitation of miRs-183, 96 and 182 by RT-qPCR analysis, and (**D**) respective reduction of miR-specific luc-3′ UTR plasmids compared to empty vector (EV) after 24 hours in RWPE2-CTRL and RWPE2-183FC cells (mean and SEM shown, n = 2). (**E**) hZIP1 gene expression normalized to HPRT in CTRL and 183FC RWPE2 cells. (**F**) 48 hour cell proliferation assay comparison between CTRL and 183FC RWPE2 cells *in vitro*. Bar graph shows mean with SEM for n = 3 experiments. (**G**) tumor volume of RWPE2-CTRL and 183FC xenografts in nude mice; n = 3 grafts for each cell type. Mean with SEM at each week. *p < 0.05 for unpaired Student’s two-sided t-test CTRL versus 183FC. (**H**) Zinc levels in the RWPE2-CTRL and 183FC tumours by Quantichrome assay (n = 3). Unless otherwise specified, mean and SEM (error bars) are shown on the graphs with statistics by paired Student’s 2-sided t-test of experimental replicates, *p < 0.05.

The effects of miR-183 cluster overexpression in PCa cells was assessed in the RWPE-2 cell line, which are syngeneic to the non-tumourigenic RWPE-1 cells, but were transformed with the Kirsten murine sarcoma virus (Ki-Ras) oncogene^21^. RWPE-2 cells have 2-fold higher levels of miR 182 compared to RWPE-1 (Fig. 4A). RWPE2-183FC and RWPE2-CTRL GFP^+^ cellular populations were generated (Fig. 4B) as described for the RWPE-1 cells. RWPE2-183FC had 5–10 fold higher levels of the mature miRs-183, -96 and -182 compared to RWPE2-CTRL (Fig. 4C). Increased miR-183 family activity was confirmed in RWPE2-183FC by significantly increased suppression of the miR-specific 3′ UTR luciferase plasmids (Fig. 4D) and reduced ZIP1 mRNA (Fig. 4E). The RWPE2-183FC cells were significantly more proliferative than the CTRL cells (Fig. 4F), a phenotype that was not observed when the miRs were overexpressed in RWPE1 cells (Supplemental Figure S2).

To test the effects of the miR-183 cluster family *in vivo*, RWPE2-CTRL or RWPE2-183FC cells were grown as subcutaneous xenografts in male NUDE mice. Whereas the RWPE2-CTRL tumours reached a maximum tumour volume of 40 mm^3^ then spontaneously regressed to 20 mm^3^, the RWPE2-183FC continued to increase in size until the study end at 13 weeks (Fig. 4G). Moreover, intra-tumoural zinc levels were significantly lower in the RWPE2-183FC grafts compared to the RWPE2-CTRL xenografts **(**Fig. 4H **)**. These experiments show that the miR-183 cluster family augments tumour growth of RWPE-2 cells and also reduces zinc *in vivo*.

### miR-183 cluster regulates cancer-related pathways

**Figure 5:**
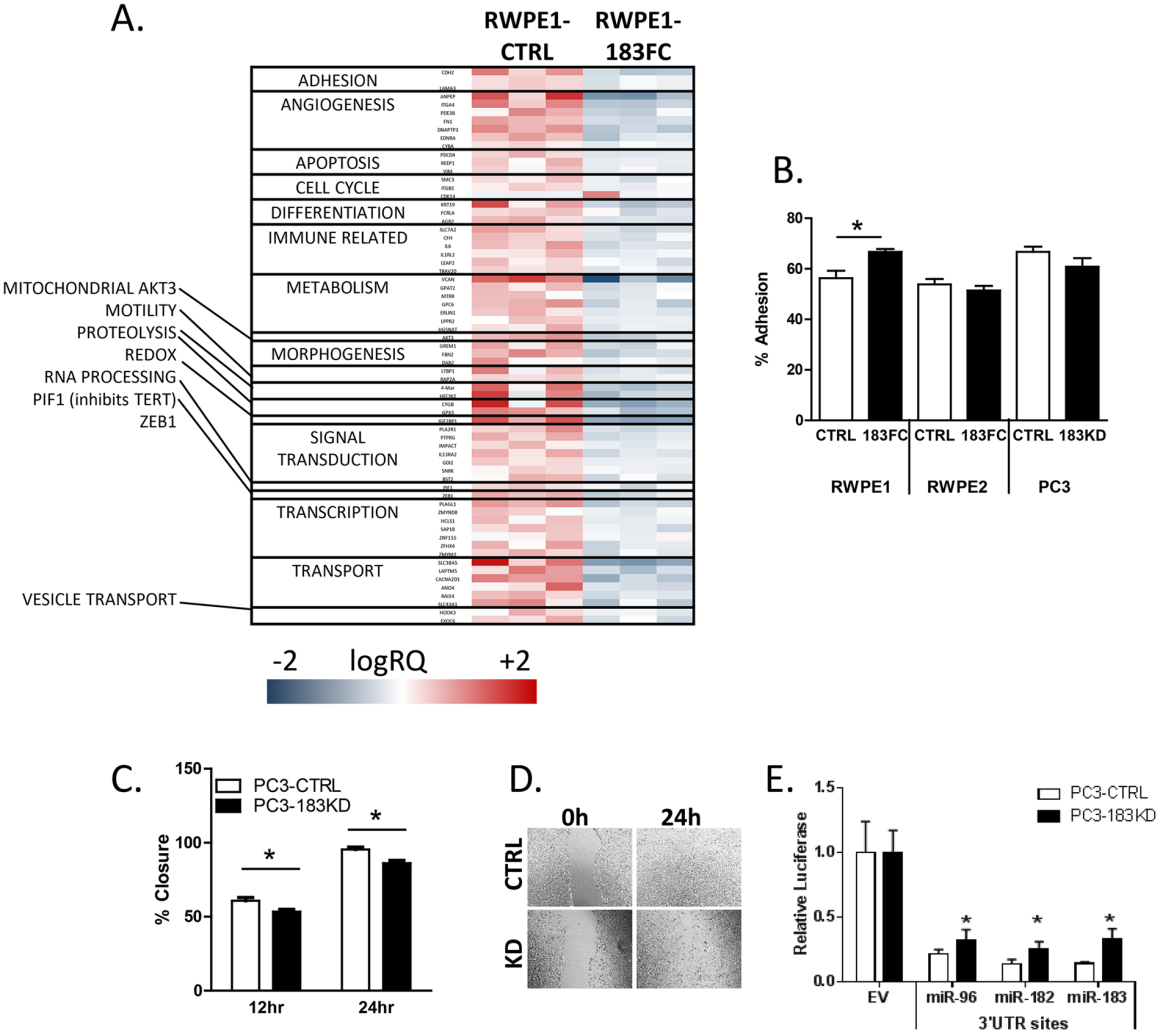
Overexpression of 183FC alters gene expression in RWPE1 and regulates motility and adhesion of prostate cells. (**A**) Heatmap of suppressed genes between RWPE1-CTRL and RWPE1-183FC cells. Affymetrix HT 1.0 Arrays were run on biological triplicates of lentivirus transduced RWPE1 cells sorted for GFP expression. Log 2 of the gene expression is shown and grouped by gene function. GSEA and DAVID Pathways are in shown in Supplemental Table 1. Full data were deposited into GEO (GSE99134). (**B**) Adhesion of cells at 30 minutes for RWPE1-CTRL, RWPE1-183FC, RWPE2-CTRL, RWPE2-183FC, PC3-CTRL and PC3-183KD. Bar graph shows mean and SEM for n = 4. (**C**) Migration of PC3-CTRL and PC3-183KD by scratch assay shown as bar graph for mean and SEM for n = 4, and, (**D**) Representative images of the scratch closure. (**E**) Respective levels of miR-specific luc-3′ UTR plasmids compared to empty vector (EV) after 24 hours in PC3-CTRL and PC3-183KD cells. Mean and SEM are shown, (n = 2). Student’s paired 2-sided t-test, *p < 0.05.

Gene expression was profiled in the RWPE1-CTRL and RWPE1-183FC by microarray. Genes significantly lower in the 183FC included those involved in adhesion, angiogenesis and metabolism (Fig. 5A). Gene set enrichment analysis and DAVID Pathway analysis showed enrichment of beta cell, RNA pol 1 promoter opening, metabolism and adhesion gene sets (Supplemental Table 1). Of note, hZIP1 (SLC39A1) was 82% lower in the 183FC compared to CTRL (data not shown), validating the RT-qPCR and immunoblot data in Fig. 2.

The phenotypic effects of the miR-183 family cluster were examined in the RWPE-1 and RWPE-2 -CTRL and -183FC cells as well as PC3 cells with reduced miR-183 family activity. PC3 cells were derived from castration resistant prostate cancer and represent highly malignant PCa cells. We previously reported that PC3 cells have the highest levels of miRs-183, 96 and 182 of the prostate cancer cell lines^18^. Thus, PC3 cells were transduced with a sequence complementary to the miR-183 family to block activity (PC3-183KD). Cell adhesion was increased in RWPE1-183FC cells compared to RWPE1-CTRL, but there was no change in the other cell lines (Fig. 5B). Cell migration was reduced in the PC3-183KD cells (Fig. 5C,D) and these cells also had decreased suppression of the miR-183 family 3′ UTRs (Fig. 5E). Migration in the RWPE-1 and RWPE-2 cells was minimal and not able to be assessed between the cell lines (data not shown).

### The intergenic region between miR-96 and -182 contains an active transcription site to regulate expression of miR-182

**Figure 6:**
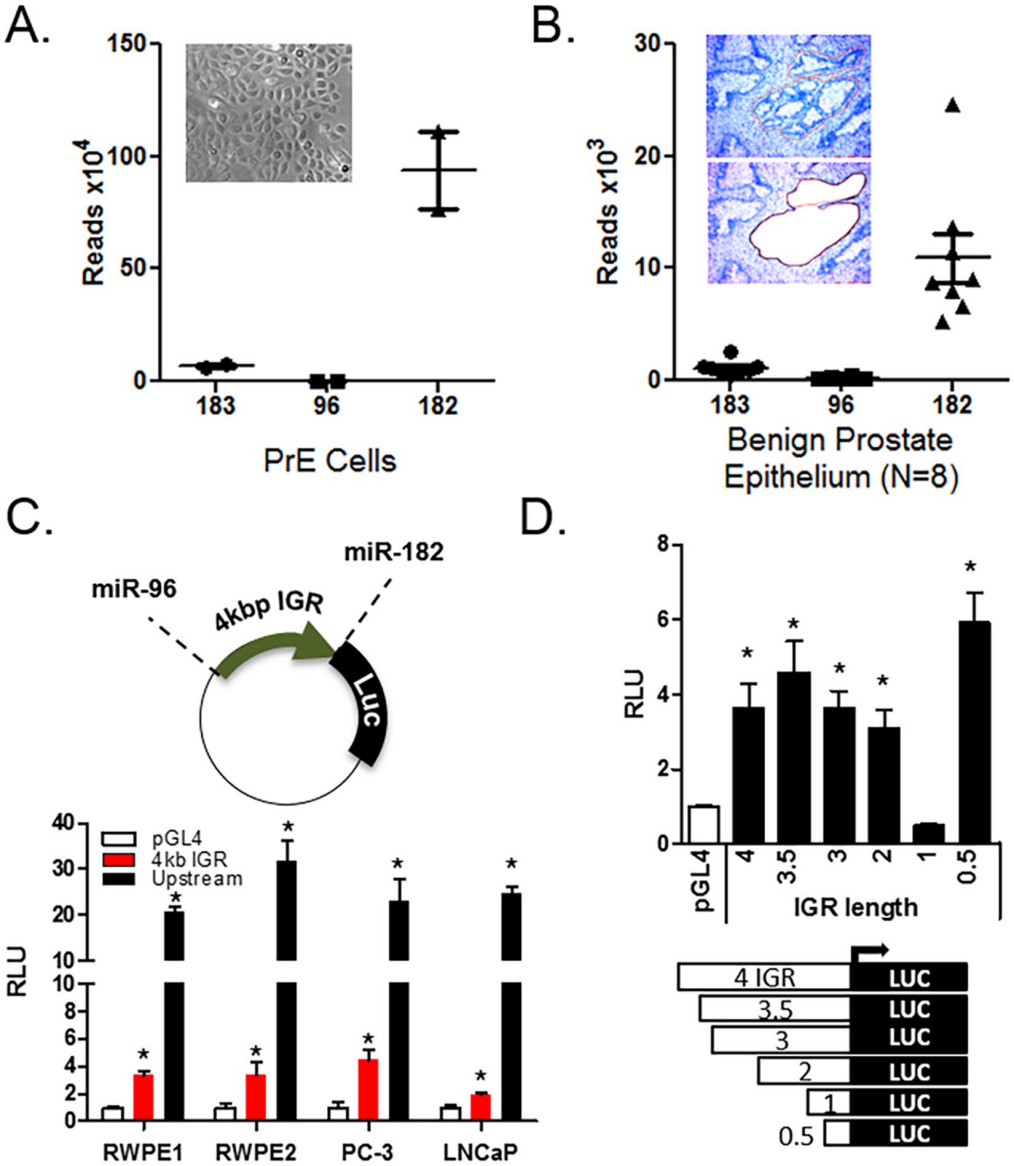
Intergenic region in the miR-183 cluster has a secondary transcriptional start site. Expression of the miR-183 family in benign prostate epithelial cells by Illumina RNAseq examined on the small RNA fraction (<50 bp) isolated from. (**A**) Primary prostate epithelial cells (PrE) (n = 2) and, (**B**) LCM-collected benign prostate epithelium from radical prostatectomy patients (n = 8). The miRs were normalized to raw reads. (**C**) Activity of a pGL4 luciferase plasmid with insertion of the intergenic region between miR-96 and -182 (IGR) as the promoter. IGR-luc in RWPE1, RWPE2, PC-3 and LNCaP prostate cell lines. pGL4 plasmid with 1 kb of the miR-183-upstream promoter included for comparison. n = 3 for each cell line. (**D**) The activity of IGR-luc deletion mutants in the RWPE1 cell line. Bar graph shows mean with SEM for n = 4. *p < 0.05 compared to pGL4 empty vector by Student’s paired 2-sided t-test.

Previous RT-qPCR data from our lab showed differential expression by RT-qPCR of the three miRs-183, -96 and -182 with miR-182 being the highest in prostate cells^18^, suggesting a differences in the mature miR transcription, processing and/or stability. As well, Figs 1C and 4C show miR-182 expressed at higher levels than the other 2 miRs by absolute quantitation^22^. We further examined the comparative expression of the miR-183 family members in PrE cells (n = 2) and laser-capture micro-dissected benign prostate epithelium (n = 8) by small RNA-sequencing (Fig. 6A,B). The RNA-sequencing data were consistent between the PrE cells and benign epithelium in showing that miR-182 was present at 10–100 fold higher levels than miRs-183 and -96 (Fig. 6A,B).

To test whether the intergenic region (IGR) between miR-96 and -182 had promoter activity that may be driving higher expression of miR-182, the IGR was cloned into pGL4 as the promoter (Fig. 6C). The IGR had significant promoter activity in all prostate cell lines examined (Fig. 6C). An −1500 bp upstream promoter has previously been reported^23^ and we cloned it into pGL4. The IGR promoter activity was 10–20% of the upstream promotor (Fig. 6C), suggesting that both promoters regulate the expression of miR-182. Targeted deletion of the IGR showed a dramatic decrease in promoter activity when the region 1–2 kb from miR-182 was deleted (Fig. 6D). Interestingly, the highest level of sequence conservation in the IGR also lies in this 1–2 kb region, supporting the presence of regulatory elements in this region that further regulate miR-182 separately from miRs-183 and -96.

## Discussion

The goal of this study was to investigate whether overexpression of the miR-183 family from its native gene cluster decreased intracellular zinc levels and altered PCa growth. Though the phenomenon of low zinc in PCa has been documented for many decades, it is still unclear whether loss of zinc contributes to prostate carcinogenesis or whether it is a bystander in the development of the tumour. The miR-183 family was previously reported by our group to be highly expressed in prostate cancers and was a regulator of zinc homeostasis^18^. However, a limitation of our prior study, and of most miR studies, was that the miRs were overexpressed by transient transfection pre-miR mimics which results in supra-physiological levels (often >1000 fold) of the mature miRs and bypasses potential regulation during pri-miR transcript processing^18^. The present study avoids the caveats of pre-miRs by cloning the entire 4.8 kb miR-183 cluster into a lentiviral vector for expression in cells to preserve the highly conserved putative regulatory elements within the 4.2 kb intergenic region between miRs-96 and -182. This method for overexpression is a more natural mimic of increased expression of all three of the miRs that is observed in PCa.

Our findings not only validated our previous work on zinc regulation, but also demonstrate that these phenotypes are observed at levels of the miR-183 family cluster that mimic those observed in tumours. The overexpression of the miRs decreased ZIP1 protein and mRNA expression in the miR-183FC transduced RWPE-1 and RWPE-2 compared to their respective controls. Importantly, zinc regulation by 183FC similarly occurred in primary epithelial prostate organoids and *in vivo* in RWPE-2 xenografts, demonstrating that the role of the miRs is not unique to one cell type or an *in vitro* phenomenon. The RWPE2-183FC tumours were also larger than controls, which consistent with other reports of overexpression of the miRs individually in PCa cell xenografts^24–27^. The decrease in zinc observed in the 183FC tumours further supports the link between zinc and PCa carcinogenesis. Since zinc depletion has been such a consistent feature in PCa, it is not clear whether the low zinc is permissive of cancer growth or is simply decreased due to epithelium dedifferentiation and loss of the zinc sequestering phenotype. Franklin and others have provided strong rationale that lower zinc does contribute to carcinogenesis due to reactivation of the Krebs cycle^1, 3, 12^.

Our ZQ data suggest that the miR-183 family members have a pronounced effect on free, loosely-bound or accessible zinc. The reduction in ZQ intensity was ~30% compared to a 15% reduction in total zinc as measured by both the Quantichrome and X-ray fluorescence. It is not possible to directly compare the assays statistically, however, the two total zinc methods (Quantichrome and XRF) had highly similar results, providing some confidence in this difference. Although this difference may seem minimal, this labile pool of free, loosely-bound or accessible zinc that is capable of affecting the cell metabolism via inhibition of aconitase as shown by Franklin and Costello^1–3^. Therefore it may be that changes in expression levels of the miR-183 family are integral for the loss of zinc observed in PCa and subsequently alters the metabolism.

Overexpression of the miR-183 family also regulated cell phenotypes such as migration, adhesion and proliferation. These phenotypes differed between benign and PCa-derived cells. In benign cells, high levels of 183FC did not alter the proliferation of RWPE-1 cells and actually reduced the size of benign PrE organoids, suggesting proliferation changes require further molecular changes. As well, since PrE cell organoids develop from progenitor cells, it is possible that the lower levels of zinc in the 183FC organoids inhibited differentiation. Gene expression analysis of RWPE1-183FC cells suggested regulatory control of several key pathways in carcinogenesis including adhesion and motility. Concordantly overexpression of 183FC enhanced adhesion in RWPE-1. Migration of all RWPE cell lines was minimal (>48 hr scratch closure), however knockdown of the miR-183 family in the more motile PC-3 cells significantly decreased their motility. In the PCa RWPE-2 cells 183FC enhanced cell proliferation *in vitro* and xenograft tumour volume *in vivo*.

The miR-183 family are considered “oncomiRs” in many other cancers and the miRs have been shown to regulate pathways dysregulated in cancer such as DNA repair, apoptosis and metabolism^28–40^. In our study, the genes with the highest magnitude of suppression were adhesion related, and this has not previously been reported. There are several potential explanations to our observation. The first is that the lowered zinc phenotype that accompanies miR-183FC overexpression is prostate cell specific, leading to repression of different target genes. Another possibility is that the coordinated expression of the miRs as a cluster (including the 4.2 kb IGR) provides a different target profile from overexpression of a single miR, and in this way our study is unique from others that have profiled gene regulation by the miRs. As further evidence of a regulatory role for the IGR, we observed promotor activity of the sequence in all prostate cell lines. Given our array findings and the promoter activity of the IGR, the role of the 4.2 kb IGR needs to be studied further as this region possesses highly conserved areas in eukaryotes that may further regulate the expression of the miRs in this cluster.

Our study adds to the understanding of miR function and regulation in prostate physiology and as a potential contributor to PCa. We have much left to learn about the function of miRs in a cell-type specific manner and transcriptional/post-transcriptional mechanisms that govern miR expression itself. Our findings may not be exclusive to PCa and could extend to other cancers that overexpress the miR-183 family. Although the zinc phenotype of PCa is well-described, it does not exclude the possibility that other tumour types have zinc disruptions. It is also important to distinguish that our work investigates the molecular changes leading to changes in cellular zinc and is not meant to advocate for dietary zinc interventions, which have had mixed results on PCa risk^41–43^.

## Methods

### Cell lines

RWPE-1, RWPE-2, PC-3 and LNCaP were obtained from American Type Culture Collection (ATCC) in 2013 and re-authenticated in 2016 (*Supplemental Document for Review purposes*). RWPE-1 and RWPE-2 cells were cultured in KSFM with bovine pituitary extract and epidermal growth factor (ThermoScientific, Waltham, MA), and used passage <21. Primary human benign epithelial cells (PrE) and organoids were grown in PrEGM media (Lonza, ThermoScientific). The PrE cells were established from fresh radical prostatectomy tissues, as previously described^19, 44^, via UIC Institutional Review Board approved protocols (#12–0331 and 15–1294). PC-3 were cultured in DMEM with 10% fetal bovine serum (FBS) and LNCaP in RPMI with 10% FBS.

### Cloning and lentiviral production

miR-183 cluster (4.8 kb) was isolated from human male DNA by nested PCR with primers F_outer_ 5′-AAGGTCATCTTGGGCTGATG-3′ and R_outer_ 5′-GAGGGTGAGAAGGGTCACCA-3′ and F_nested_ 5′-GACTGAAGTCGGGGGTAGAGACCGTAGCAG-3′ and R_nested_ 5′-CTGACTTCAGGGATGCAGGGAAACACAGAG-3′. This PCR product (miR-183FC) was cloned into an intermediate vector and sub-cloned using primers with NheI sites: 5′ ATTCGAATTC**GCTAGC** **T** GGCTGTGCACAGGGTGC 3′ and 5′ AGATTCTAG**AGCTAGC**GGGAACGGGCATCGTGGG 3′. miR-183FC was cloned into lentiviral vector CD511B-1 (Systems Biosciences, Mountain View, CA) with NheI enzyme (New England Biolabs, Ipswich, MA) and propogated in Stellar Competent Cells (Clontech, Mountain View, CA). Clones were verified by sequencing.

### Lentiviral transduction

Ultrahigh titre of lentivirus from CD511B-1 + miR-183FC and CD511B-1 scrambled control was produced by Systems Biosciences (Mountain View, CA) and used to transduce RWPE-1, RWPE-2, and PrE cells by spinfection^45^ via UIC Institutional Biosafety Committee approved protocol (#15–020). Briefly, lentivirus (120 MOI) and polybrene (8 μg/ml) (Sigma Aldrich St. Louis, MO) were added to 75,000 cells in a polystyrene tube, incubated 1hr 37 °C, centrifuged at 750 × g 25 °C for 1hr and re-plated. GFP expression was evident at 72hr using EVOS fluorescence microscope (ThermoScientific). PC-3 with knockdown of the miR-183 family were generated using the miRZIP Lentivector-based Anti-miR system (System Biosciences, Palo Alto, CA), with shRNA targeted to miR-183 or control scrambled shRNA, and maintained under selection with 1.5 μg/ml puromycin.

### Sorting of GFP^+^ population of cells

The transduced RWPE cells were GFP^+^ cells were sorted on MoFlo Astrios (Beckman Coulter, Brea, CA) in 1 ml of FACS sorting buffer (1X PBS +0.5% BSA +1 mM EDTA) based on GFP detection by 488 nm laser. Sorted cells were collected into collection buffer (1X complete KSFM +1X Ab/Am +30% FBS), washed and plated to expand the cultures.

### miR and mRNA expression

RNA was isolated from cells at 70% confluency using Trizol (ThermoScientific) with the small RNA recovery protocol. RNA quantity and quality was determined with the NanoDrop Spectrophotometer (Thermo Scientific, Waltham, MA) by absorbance at 230, 260 and 280 nm. For miR RT, 150 ng of RNA was converted to cDNA using the Exiqon Universal cDNA kit (Exiqon, Denmark). For the mRNA-RT, 355 ng of RNA was converted to cDNA using High Capacity Reverse Transcription Kit (ThermoScientific). SYBR real-time RT-qPCR was run on the QuantStudio6 (ThermoScientific). C_T_ values were normalised to housekeeping RNAs (as indicated in the figure legends) by the ΔΔC_T_ method^46^.

For absolute quantitation, RNA oligos with 5′ phosphorylation for miR-183, -96 and -182 (IDT) were used as an RT template to generate a standard curve to calculate the number of miRs per ng of total RNA in the reaction. RNU44 was used to normalize for input.

### Luciferase-3′ UTR for miR activity

LightSwitch-RenSP vectors from SwitchGear Genomics (Carlsbad, CA, US) for control (3′ UTR with no miR consensus sites) and miRs-96, -182 and 183 (miR-specific sites in 3′ UTR) were used. Cells were plated at 30 K (RWPE) or 50 K (PC-3) cells/well in a 24-well plate, grown overnight, then transfected with 100ng of LightSwitch vector and empty pGL4-luc control vector with DharmaFECT (Pittsburgh, PA, USA). Renilla and luciferase activity were assayed in triplicate 24 h after transfection with Dual-luciferase assay (Promega, Madison, WI, USA). 3′ UTR levels were normalised to the LightSwitch control vector for each cell type.

### Immunoblot

Cells were scraped into 75 µl of 1X protein lysis buffer (Cell Signaling, Danvers, MA, USA). Insoluble fraction was pelleted at 10000 × g for 10 minutes (4 °C). Thirty µg of protein was separated on a 4–12% Bis-Tris NuPage gel (Thermo Scientific) and transferred to PVDF membrane. ZIP1 chicken polyclonal antibody (gift from Dr. Renty Franklin) was used at 1:10000 and β-tubulin rabbit antibody at 1:1000 4 °C overnight. Secondary anti-chicken or anti-rabbit (Li-cor, Lincoln, NE) was used at 1:1000 for 1 hour at room temperature and was analysed on the Li-cor Odyssey system.

### Total intracellular zinc

Cells were harvested into zinc-depleted hypotonic buffer (10 mM Tris, 10 mM NaCl, 3 mM MgCl_2_, 1% NP-40, 10 mM PMSF chelated with chelex). For subcutaneous tumours, the frozen tissues were homogenised in 200 µl of chelated hypotonic buffer. Cell and tissue samples were sonicated, centrifuged at 10000xg (4 °C) and the supernatant assayed with the Quantichrome Zinc Assay kit (BioAssay Systems, Hayward, CA). Zinc was normalised to total protein which was determined by Biorad Protein Assay (Bio-Rad, Hercules, CA).

### Zinquin fluorescence of free and loosely bound zinc

Naïve RWPE-1 cells were mixed 1:1 with GFP^+^ RWPE1-CTRL or RWPE1-183FC and allowed to attach overnight. Zinquin (20 µM)(Santa Cruz Biotechnology, Dallas, TX) was added and incubated for 20 minutes at 37 °C. Cells were imaged using confocal microscopy (Zeiss, Ontario, CA) and the EVOS FL Cell Imaging System (ThermoFisher, USA). GFP^+^ cells were compared to GFP^−^ naïve cells with inForm Cell Analysis Software (Perkin Elmer, USA) followed by Mann-Whitney U nonparametric test of the values using GraphPad Prism.

### Gene expression profiling of 183C target genes

RNA (2 µg) from RWPE1-CTRL and RWPE1-183FC biologic triplicates were labelled and hybridised to GeneChip® Human Gene 1.0 ST Arrays (Affymetrix, Santa Clara, CA, USA). Raw cel files were deposited into GEO (GSE99134) *Analysis*. RMA normalization was performed for the raw CEL files using the R Bioconductor “oligo” package^47^. The well annotated genes were kept using the annotation file from Affymetrix (HuGene-2_0-st). GSEA^48^ was used to identify the gene sets (pathways) associated with RWPE1-CTRL and RWPE1-183FC using the curated gene sets (C2) from molecular signature database (MSigDB).

To identify the differentially expressed genes (DEGs), genes with median expression levels <the 20th percentile of medians for all genes or with variance <the 20th percentile of variances for genes were removed. The R Bioconductor “limma” package^49^ was used to obtain the top differentially expressed genes between RWPE1-CTRL and RWPE1-183FC, using a false-discovery rate (FDR) of less than 0.2 and absolute fold change greater than 1.5 as thresholds. Pathway annotation for the list of differentially expressed genes was done with the “Functional Annotation” module in the Database for Annotation, Visualization and Integrated Discovery (DAVID)^50^.

### Zinc measurement by X-Ray fluorescence of primary prostate organoids and prostate tissues

Benign PrE cells were derived from radical prostatectomy patients via an Institutional Review Board (IRB)– approved human subjects Biorepository protocol with informed consent as previously described^44, 51, 52^. All experiments with human cells and tissues were performed in accordance with the human subjects’ guidelines and regulations in a de-identified manner. Benign PrE cells were transduced with CTRL or 183FC by spinfection and plated in growth factor reduced matrigel® (Corning, USA) 1:1 with PrEGM. After 14 days, intact organoids were removed with dispase (1 U/mL) and fixed in 4% paraformaldehyde. The organoids were visualised on the EVOS FL Cell Imaging System and GFP^+^ organoids were collected by pipet. Organoids were placed in histogel and paraffin-embedded. Sections (10 µm) were placed onto 2 mm × 2 mm silicon nitride membrane windows (Silson Ltd, Northampton, England).

Prostate tissues were acquired from radical prostatectomy patients via the same IRB-approved biorepository protocol with consent. Fresh tissues were fixed in 4% paraformaldehyde, paraffin-embedded and sectioned onto the windows. X-ray fluorescence data were collected at the X-ray Science Division beamlines at the Advanced Photon Source Beamline 2I-DE, Argonne National Laboratory (Lemont, IL). Data visualization and quantitation of regions of interest (ROI) was performed with MapsSy (Stefon Vogt, Argonne National Lab). Three ROI were drawn for each organoid, the background was subtracted and the mean μg/cm^2^ for zinc was normalised to the mean sulfur for each slide. Data from all organoids (six CTRL and four 183FC) from two different patients were included in student’s t-test analysis.

### RWPE-2 subcutaneous grafts

The animal experimental protocol (#12–076) was approved by the Institutional Animal Care and Use Committee (IACUC) at UIC, also known as the Animal Care Committee (ACC). All experiments were performed in accordance with US federal guidelines for animal care. RWPE2-CTRL or RWPE2-183FC (7.5 × 10^5^ cells) were mixed 1:1 with matrigel® and injected into the flank of 17 week old athymic foxn/nu nude mice (Harlan, Indianapolis, IN). A silastic testosterone implant (1 cm) was placed subcutaneously in the mice as previously described by Hu *et al*.^53^. Tumour size was measured weekly using calipers. Tumour volumes were calculated using the following formula: ½ × (Length × Width × Width). Mice were euthanised and the tumours were snap frozen in liquid nitrogen for zinc measurements.

### Small RNA sequencing of PrE cells and benign prostate epithelium

RNA was collected from PrE cells as described above. For the tissues, RNA was isolated with RNAqueous®-Micro kit (Ambion, USA) from benign prostate epithelium which was collected by laser-capture micro-dissection from eight de-identified frozen prostatectomy specimens (UI Biorepository)^54–56^. RNA quality was assessed by the Bioanalyzer (Agilent) and libraries were constructed with 500 ng of RNA using the TruSeq Small RNA Sample Prep Kit (Illumina, USA). The libraries were quantitated by qPCR and sequenced 8/lane for 51 cycles on a HiSeq. 2500 using TruSeq Rapid SBS sequencing chemistry v2. Fastq files were generated and demultiplexed with the bcl2fastq v1.8.4 Conversion Software (Illumina). The number of reads per patient ranged from 14 to 21 × 10^6^. *Analysis*. Adapters were trimmed, the reads reduced and mature miRs were mapped to hg19. Number of reads for each miR is shown as a sum of all isomers and normalised to raw reads.

### Luciferase Assay for promoter activity

The intergenic region between miRs-96 and -182 was isolated by PCR from gDNA with primers F-GAGACTCGAGGGAGCCCAGCAATCTGAG and R-TCTCAAGCTTCTCGGTCTGTGCTGAGGAA. The region was cloned in pGL4 (Promega, Madison, WI, USA) and verified by sequencing as pGL4-IGR. Deletion mutants of with various lengths of promoter upstream of miR-182 (3.5 kb: 3,524 bp; 3 kb: 3,014 bp; 2 kb: 2,021 bp; 1 kb: 1,059 bp and 5 kb: 613 bp) were cloned into pGL4 (Creative Biogene, Shirley, NY). Cells were plated in a 24-well plate in triplicate and transfected using DharmaFect with pGL4 empty vector or pGL4-IGR and prL-null (Promega) as a control. Luciferase and renilla values were measured using the Dual-luciferase® Reporter Assay System (Promega) on the Glomax 20/20 (Promega). Luciferase relative light units (RLU) were normalised to renilla for transfection and cell number control.

### Migration assay

Cells were grown to confluence in a 24-well plate (3 replicates), then scratched with a Falcon 100 µl pipette tip down the centre of each well. Images of the same location in each scratch were taken using the EVOS FL Auto (Thermo Scientific) at times 0 hr, 12 hr and 24 hr post-scratch. Scratch area was measured using inForm 2.2 software (Perkin Elmer).

### Adhesion assay

Cells in media containing NucBlue Live Reagent (ThermoScientific) were plated at 5,000 cell/well in a 96-well plate for 15 m, 30 m, 45 m and 1hr total (3 replicates each) before the plate was inverted and tapped to remove media from the wells. Adherent cells were imaged using the EVOS FL Auto (ThermoScientific). As a control for total cells plated, separate wells were imaged at 3hr without media removal. Cell counts were generated using inForm 2.2 software (Perkin Elmer).

### Statistics

Statistics were performed using GraphPad Prism 5.04. Most data were normally distributed and were analyzed by Student’s 2-sided t-test; paired for experimental replicates and unpaired for xenograft study. Relative expression by qPCR and Zinquin fluorescence were not normally distributed and were analyzed by Mann-Whitney non-parametric test. p < 0.05 was considered significant. Experiment-specific details are in the figure legends.

## Electronic supplementary material

Supplementary Information

## References

[1] Costello LC, Franklin RB (2006). The clinical relevance of the metabolism of prostate cancer; zinc and tumor suppression: connecting the dots. Molecular cancer.

[2] Guan Z (2003). Kinetic identification of a mitochondrial zinc uptake transport process in prostate cells. Journal of Inorganic Biochemistry.

[3] Costello LC, Liu Y, Franklin RB, Kennedy MC (1997). Zinc inhibition of mitochondrial aconitase and its importance in citrate metabolism of prostate epithelial cells. Journal of Biological Chemistry.

[4] Chyan W, Zhang DY, Lippard SJ, Radford RJ (2014). Reaction-based fluorescent sensor for investigating mobile Zn2+ in mitochondria of healthy versus cancerous prostate cells. Proceedings of the National Academy of Sciences of the United States of America.

[5] Franklin RB (2005). hZIP1 zinc uptake transporter down regulation and zinc depletion in prostate cancer. Molecular cancer.

[6] Kim JK (1998). *In vivo* differential diagnosis of prostate cancer and benign prostatic hyperplasia: localized proton magnetic resonance spectroscopy using external-body surface coil. Magnetic Resonance Imaging.

[7] Kavanagh JP, Darby C, Costello CB (1982). The response of seven prostatic fluid components to prostatic disease. International Journal of Andrology.

[8] Kambe T, Hashimoto A, Fujimoto S (2014). Current understanding of ZIP and ZnT zinc transporters in human health and diseases. Cellular and Molecular Life Sciences.

[9] Kambe T, Tsuji T, Hashimoto A, Itsumura N (2015). The Physiological, Biochemical, and Molecular Roles of Zinc Transporters in Zinc Homeostasis and Metabolism. Physiological Reviews.

[10] Xie J (2015). Zinc inhibits Hedgehog autoprocessing: linking zinc deficiency with Hedgehog activation. Journal of Biological Chemistry.

[11] Truong-Tran AQ, Carter J, Ruffin RE, Zalewski PD (2001). The role of zinc in caspase activation and apoptotic cell death. Biometals.

[12] Liang JY (1999). Inhibitory effect of zinc on human prostatic carcinoma cell growth. Prostate.

[13] Feng P, Li T, Guan Z, Franklin RB, Costello LC (2008). The involvement of Bax in zinc-induced mitochondrial apoptogenesis in malignant prostate cells. Molecular cancer.

[14] Kolenko V, Teper E, Kutikov A, Uzzo R (2013). Zinc and zinc transporters in prostate carcinogenesis. Nat Rev Urol.

[15] Franklin RB (2003). Human ZIP1 is a major zinc uptake transporter for the accumulation of zinc in prostate cells. Journal of Inorganic Biochemistry.

[16] Huang L, Kirschke CP, Zhang Y (2006). Decreased intracellular zinc in human tumorigenic prostate epithelial cells: a possible role in prostate cancer progression. Cancer Cell Int.

[17] Golovine K (2008). Overexpression of the zinc uptake transporter hZIP1 inhibits nuclear factor-kappaB and reduces the malignant potential of prostate cancer cells *in vitro* and *in vivo*. Clinical Cancer Research.

[18] Mihelich BL (2011). miR-183-96-182 cluster is overexpressed in prostate tissue and regulates zinc homeostasis in prostate cells. Journal of Biological Chemistry.

[19] Giangreco AA (2015). Differential expression and regulation of vitamin D hydroxylases and inflammatory genes in prostate stroma and epithelium by 1,25-dihydroxyvitamin D in men with prostate cancer and an *in vitro* model. The Journal of steroid biochemistry and molecular biology.

[20] Zalewski P (2006). Use of a zinc fluorophore to measure labile pools of zinc in body fluids and cell-conditioned media. Biotechniques.

[21] Bello D, Webber MM, Kleinman HK, Wartinger DD, Rhim JS (1997). Androgen responsive adult human prostatic epithelial cell lines immortalized by human papillomavirus 18. Carcinogenesis.

[22] Ferre F (1992). Quantitative or semi-quantitative PCR: reality versus myth. PCR Methods Appl.

[23] Li P (2014). MiR-183/-96/-182 cluster is up-regulated in most breast cancers and increases cell proliferation and migration. Breast Cancer Res.

[24] Hirata H (2013). MicroRNA-182-5p promotes cell invasion and proliferation by down regulating FOXF2, RECK and MTSS1 genes in human prostate cancer. PloS one.

[25] Li Y (2015). Hypoxia-inducible miR-182 enhances HIF1alpha signaling via targeting PHD2 and FIH1 in prostate cancer. Sci Rep.

[26] Ma Y (2014). Biphasic regulation of autophagy by miR-96 in prostate cancer cells under hypoxia. Oncotarget.

[27] Ueno K (2013). microRNA-183 is an oncogene targeting Dkk-3 and SMAD4 in prostate cancer. Br J Cancer.

[28] Shi XY, Gu L, Chen J, Guo XR, Shi YL (2014). Downregulation of miR-183 inhibits apoptosis and enhances the invasive potential of endometrial stromal cells in endometriosis. International journal of molecular medicine.

[29] Wang YQ, Guo RD, Guo RM, Sheng W, Yin LR (2013). MicroRNA-182 promotes cell growth, invasion, and chemoresistance by targeting programmed cell death 4 (PDCD4) in human ovarian carcinomas. Journal of cellular biochemistry.

[30] Tang H (2013). The miR-183/96/182 cluster regulates oxidative apoptosis and sensitizes cells to chemotherapy in gliomas. Current cancer drug targets.

[31] Fendler A (2013). The antiapoptotic function of miR-96 in prostate cancer by inhibition of FOXO1. PloS one.

[32] Krishnan K (2013). MicroRNA-182-5p targets a network of genes involved in DNA repair. RNA.

[33] Weeraratne SD (2012). Pleiotropic effects of miR-183~96~182 converge to regulate cell survival, proliferation and migration in medulloblastoma. Acta neuropathologica.

[34] Wang Y, Huang JW, Calses P, Kemp CJ, Taniguchi T (2012). MiR-96 downregulates REV1 and RAD51 to promote cellular sensitivity to cisplatin and PARP inhibition. Cancer research.

[35] Liu Z (2012). MiR-182 overexpression in tumourigenesis of high-grade serous ovarian carcinoma. J Pathol.

[36] Yao E, Ventura A (2011). A new role for miR-182 in DNA repair. Mol Cell.

[37] Roy R, Chun J, Powell SN (2011). BRCA1 and BRCA2: different roles in a common pathway of genome protection. Nat Rev Cancer.

[38] Moskwa P (2011). miR-182-mediated downregulation of BRCA1 impacts DNA repair and sensitivity to PARP inhibitors. Molecular cell.

[39] Tanaka H (2013). MicroRNA-183 upregulates HIF-1alpha by targeting isocitrate dehydrogenase 2 (IDH2) in glioma cells. Journal of neuro-oncology.

[40] Vohwinkel CU (2011). Elevated CO(2) levels cause mitochondrial dysfunction and impair cell proliferation. Journal of Biological Chemistry.

[41] Epstein MM (2011). Dietary zinc and prostate cancer survival in a Swedish cohort. Am J Clin Nutr.

[42] Park SY, Wilkens LR, Morris JS, Henderson BE, Kolonel LN (2013). Serum zinc and prostate cancer risk in a nested case-control study: The multiethnic cohort. Prostate.

[43] Prasad AS, Beck FW, Snell DC, Kucuk O (2009). Zinc in cancer prevention. Nutr Cancer.

[44] Giangreco AA (2013). Tumor suppressor microRNAs, miR-100 and -125b, are regulated by 1,25-dihydroxyvitamin D in primary prostate cells and in patient tissue. Cancer prevention research.

[45] Goldstein AS (2011). Purification and direct transformation of epithelial progenitor cells from primary human prostate. Nature Protocols.

[46] Livak KJ, Schmittgen TD (2001). Analysis of relative gene expression data using real-time quantitative PCR and the 2(-Delta Delta C(T)) Method. Methods.

[47] Carvalho BS, Irizarry RA (2010). A framework for oligonucleotide microarray preprocessing. Bioinformatics.

[48] Subramanian A (2005). Gene set enrichment analysis: a knowledge-based approach for interpreting genome-wide expression profiles. Proc Natl Acad Sci USA.

[49] Smyth, G. K. Linear models and empirical bayes methods for assessing differential expression in microarray experiments. *Stat Appl Genet Mol Biol* **3**, Article3, doi:10.2202/1544-6115.1027 (2004). 10.2202/1544-6115.1027 16646809

[50] Huang DW (2007). DAVID Bioinformatics Resources: expanded annotation database and novel algorithms to better extract biology from large gene lists. Nucleic Acids Res.

[51] Mihelich BL (2011). miR-183-96-182 cluster is overexpressed in prostate tissue and regulates zinc homeostasis in prostate cells. The Journal of biological chemistry.

[52] Peehl DM (2005). Primary cell cultures as models of prostate cancer development. Endocr Relat Cancer.

[53] Hu WY (2011). Estrogen-initiated transformation of prostate epithelium derived from normal human prostate stem-progenitor cells. Endocrinology.

[54] Lugli, G. *et al*. Laser-capture Microdissection of Human Prostatic Epithelium for RNA Analysis. *Journal of visualized experiments: JoVE*, doi:10.3791/53405 (2015). 10.3791/53405 PMC4692752 26651078

[55] Nonn L, Vaishnav A, Gallagher L, Gann PH (2010). mRNA and micro-RNA expression analysis in laser-capture microdissected prostate biopsies: valuable tool for risk assessment and prevention trials. Experimental and molecular pathology.

[56] Richards Z (2017). Prostatic compensation of the vitamin D axis in African American men. JCI Insight.

