# Supplementary material for: The miR-183 family cluster alters zinc homeostasis in benign prostate cells, organoids and prostate cancer xenografts
Source: Sci Rep. 2017 Aug 9;7:7704. doi: 10.1038/s41598-017-07979-y (PMC5550464; doi:10.1038/s41598-017-07979-y)
Supplement: Supplementary file 1 — Supplementary Information [file 41598_2017_7979_MOESM1_ESM.pdf]

The miR-183 family cluster alters zinc homeostasis in benign prostate cells, organoids and prostate cancer xenografts

Shweta Dambal[1#], Bethany Baumann[1#], Tara McCray[1], LaTanya Williams[1], Zachary Richards[1], Ryan Deaton[1], Gail S Prins[1,2,3], Larisa Nonn[1,3,*]

[1] Department of Pathology, University of Illinois at Chicago, Chicago, IL, 60612

[2] Department of Urology, University of Illinois at Chicago, Chicago, IL, 60612

[3] University of Illinois Cancer Center, Chicago, IL 60612

[#]both authors contributed equally

*corresponding author, correspondence to lnonn@uic.edu

Supplemental Table1. GSEA and DAVID Pathway Analyses of gene expression in RWPE1-CTRL versus RWPE1-183FC

Gene Set Enrichment Analysis CTRL versus 183FC (GSEA)[1]

Gene set	# Genes	ES[2]	p-value[3]	q-value[4]
REACTOME: beta cells	19	-0.749	0	0.04
REACTOME: RNA pol1 promoter opening	43	-0.588	0	0.08
KEGG: glutathione metabolism	49	-0.558	0	0.14
REACTOME:_meiotic recombination	67	-0.497	0	0.20

DAVID Pathway Analysis[5]

Term	# Genes	FE[6]	p-value[3]	q-value[4]
KEGG: Focal Adhesion	5/11	11.5	0.0004	0.01
KEGG: ECM-receptor interaction	4/11	22.0	0.0004	0.02

[1]GSEA using the curated gene sets (C2)
[2]ES=Enrichment Score
[3]Nominal p-value
[4]q-value (FDR, false discovery rate)
[5]DAVID Pathway analysis using KEGG annotation
[6]FE=Fold Enrichment

A.

Transduce PrE cells with 183 lentivirus

↓

Grow in 3D matrigel 14 days

↓

Dispase out of matrigel

↓

Fix in PFA

↓

Pick GFP+ organoids by hand with pipet

↓

Embed into histogel "puck"

↓

Embed into paraffin

↓

Section onto SiN_2 window

↓

X-Ray image at APS (12h per window)

↓

IHC for GFP on SiN_2 window

B.

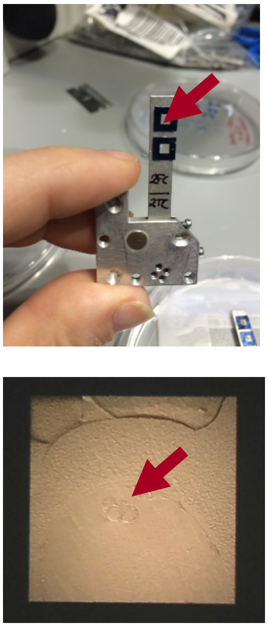

C.

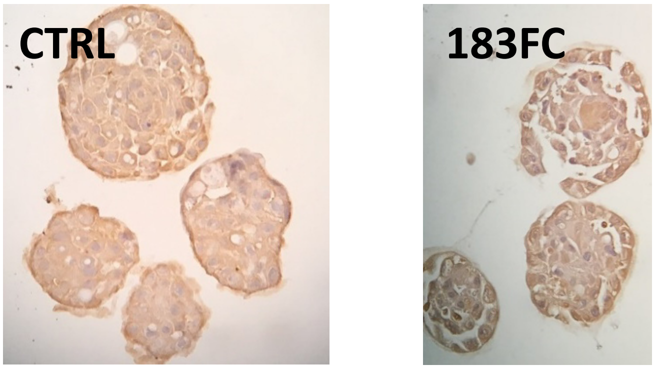

Supplemental Figure 1. **X-ray fluorescence at the Advanced Photon Source (APS) of Argonne National Labs. A**, steps in the methodology for the experiments. **B**, image of the silcon nitride (SiN_2) windows used to mount organoids for the beam Arrows indicate location of the organoids. **C**, Overexpression of CTRL and 183FC confirmed by immunohistochemical (IHC) staining with anit-GFP in day 14 benign human prostate epithelial organoids. These organoids are also shown in **Figure 3** imaged by X-ray fluorescence .

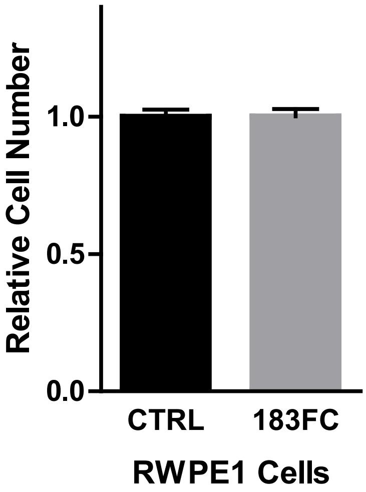

Supplemental Figure 2. Cell proliferation of RWPE1 cells overexpressing miR-183-full cluster (183FC) did not differ from control-GFP (CTRL) cells. 48 hour cell number determined by MTS assay. Non-significant by unpaired student's t-test. Mean of three experiments with SEM shown.
